# Poor and Lost Connections: Essential Family Caregivers’ Experiences Using Technology with Family Living in Long-Term Care Homes during COVID-19

**DOI:** 10.1177/07334648221081850

**Published:** 2022-04-13

**Authors:** Charlene H. Chu, Amanda Yee, Vivian Stamatopoulos

**Affiliations:** 1 70379 Lawrence S. Bloomberg Faculty of Nursing, University of Toronto, Toronto, ON, Canada; 2 Institute of Institute for Life Course & Aging, 70379 University of Toronto, Toronto, ON, Canada; 3 KITE, Toronto Rehabilitation Institute, University Health Network, Toronto, ON, Canada; 4 Faculty of Arts and Science, 70379 University of Toronto, Toronto, ON, Canada; 5 Faculty of Social Science and Humanities, 85458 Ontario Tech University, Oshawa, ON, Canada

**Keywords:** Nursing homes, technology, family caregivers, long-term care homes, COVID-19

## Abstract

**Background:** Long-term care homes (LTCHs) restricted essential family caregivers’ (EFCs) visitations during COVID-19, and virtual visits using technology were used. **Objective:** To understand EFCs’ virtual visitations experiences during COVID-19 in two Canadian provinces. **Methods:** Seven focus groups were conducted with EFCs. Thematic analysis was used to identify themes at micro, meso, and macro levels. **Results:** Four themes were found: 1) a lack of technology and infrastructure; 2) barriers to scheduling visitations; 3) unsuitable technology implementation; and 4) inability of technology to adapt to residents’ needs. **Discussion:** Virtual visitations showcased a confluence of micro, meso, and macro factors that, in some cases, negatively impacted the EFCs, residents, and the relationship between EFCs and residents. Structural and home inequities within and beyond the LTCH impacted the quality of technology-based visitations, underscoring the need to support technology infrastructure and training to ensure residents are able to maintain relationships during visitation bans. **Conclusion:** EFCs’ experiences of technology-based visitations were impacted by structural vulnerabilities of the LTCH sector.

## Background

Globally, the COVID-19 pandemic exacted unparalleled devastation on older people living in residential long-term care homes (LTCHs) and on those who care for them (Comas-Herrera et al., 2021; McGilton et al., 2020; Mendenhall, 2020). By February 2021, data from 22 countries showed that 46% of deaths attributed to COVID-19 were people living in residential LTCHs, despite LTCH residents representing roughly five percent of the population in those countries (Comas-Herrera et al., 2021). Older adult residents are at a high risk of functional and cognitive decline within short periods of time (Chu, Quan, Gandhi, and McGilton, 2021). To curb the transmission of COVID-19 and protect LTCH residents, many countries including Canada, U.S., Switzerland, China, and Japan had imposed strict public health measures including visitor restrictions that banned non-essential visitors from entering the homes (Centers for Medicare & Medicaid Services, 2020; Chu, Quan, and McGilton, 2021; Shi et al., 2020). Across Canada, the Chief Medical Officers of Health in all the provinces began issuing memorandums regarding these restrictions in March 2020 (Hinshaw, 2020; Williams, 2020; Woo, 2020). Alongside these restrictive protective measures, the Federal government implemented further restrictions such as discontinuation of communal dining and recreational activities (Government of Canada, 2021).

LTCHs are facillities that offer 24-hour nursing care to assist adult residents with activities of daily living (Government of Ontario, 2014). The ownership of publicly funded LTCHs offering 24-hour nursing care can be either *public* or *private*, whereas privately owned LTCHs can be subdivided into *for-profit* and *not-for-profit* organizations (Canadian Institute for Health Information, 2021). Within LTCHs, essential family caregivers (EFCs) play a critical multi-dimensional role encompassing physical support, socio-emotional care, care coordination, and advocacy of the residents’ concerns (Puurveen et al., 2018). They also serve as substitute decision-makers when the care recipient loses the capacity to make important decisions (National Academies of Sciences Engineering and Medicine, 2016). More notably, alongside being unpaid (Armstrong et al., 2020), “invisible” and underappreciated (Baumbusch & Phinney, 2014), caregivers typically balance work obligations, which can strain their physical and mental health (Duxbury et al., 2009). This is especially true for women as they commonly take on these roles (National Partnership for Women & Families, 2018).

During the COVID-19 pandemic, EFCs were more important than ever (Tupper et al., 2020) given the consequences of the visitor restrictions including decreased resident well-being and quality of life (O’Caoimh et al., 2020; Stall et al., 2020), long periods of social isolation (Chu et al., 2020; Chu, Wang et al., 2021), and diminished informal care provided by families (Avidor & Ayalon, 2021). Virtual visits for older adults have been utilized to facilitate virtual care during COVID-19 (Franzosa et al., 2021) and as a means to connect residents to family and friends (Sacco et al., 2020). Despite the use of technology-based visits, there are barriers related to technology use among older adults in LTCHs, which have made its application challenging (Chu, Ronquillo et al., 2021). For instance, the lack of affordable devices and internet services, older adults’ lack of confidence and digital literacy to navigate the use of the technology, and the technologies are not designed with older people in mind may deter their use (AGE-WELL National Innovation Hub, 2018; Barnard et al., 2013; Chu, Nyrup et al., 2021, Damodaran et al., 2014; Franzosa et al., 2021; Martins Van Jaarsveld, 2020; Vaportzis et al., 2017; Vassli & Farshchian, 2018). Another challenge is the poor digital infrastructure of LTCHs to support technology use, such as limited internet connections that are often in outdated physical buildings and a lack of technology support for staff (Chu, Ronquillo et al., 2021; Moyle et al., 2018). Yet despite these long-standing structural barriers, LTCHs promoted the use of technology visits as the primary means of communication to EFCs. Little is known about EFCs’ perspectives of using technology to communicate with their loved ones in LTCHs with restrictive visitation policies in place. This study aims to explore the experiences of EFCs using technology to maintain connections with LTCH residents during the COVID-19 pandemic.

## Methods

This paper is based on a larger mixed-methods study that sought to explore the lived experiences of residents and their EFCs who were restricted access to their loved ones in LTCHs due to COVID-19 policies including their experiences with visitation. This study and all study materials were approved by the Research Ethics Boards at the University of Toronto (REB #40070) and Ontario Tech University (REB #16086). Participants’ names and contact information were stored separately from their responses to ensure confidentiality.

### Participant Recruitment

Caregivers who identified as EFCs were recruited on social media (Twitter) through the principal investigators' (PIs) professional accounts. Eligible participants needed to meet the following inclusion criteria: 1) they were family members of loved one(s) living in an LTCH and were restricted access to their loved one(s) in LTCH due to policies related to COVID-19; 2) able to speak and understand English; 3) able to provide informed consent; 4) lived in Canada; and 5) have internet access. Purposive sampling was used to recruit EFCs and efforts were made to get an equal sample of male and female caregivers; however, the majority (96.7%) were female. The PIs had no piror relationships with the participants but some individuals may have been familiar with the PIs’ advocacy efforts. Participants who met the criteria and interested reached out to the PIs via the email address provided on the Twitter recruitment post. The researchers then sent interested participants a electronic consent form prior to study-related activities.

### Data Collection

Seven caregiver focus groups were conducted online through Zoom between January 2021 and March 2021. Prior to the focus groups, caregivers filled out a demographic survey and responded to questions and statements regarding their visits, caregiving role, and loved ones' quality of life. By this time, caregivers had experienced nearly a year of the pandemic with strict lockdown policies in LTCHs. Focus groups comprised of four to five caregivers were moderated by VS and lasted approximately 90-minutes. A piloted, semi-structured interview guide was used to explore participants’ experiences of socially connecting with their loved one(s) residing in LTCHs during the pandemic and the barriers and facilitators to maintaining these relationships using technology in lieu of their usual pre-pandemic, unrestricted, in-person visitation. With consent, videos of the focus groups were recorded for preparing transcripts from the discussions. The videos also informed the transcripts with observational notes that otherwise may not be captured by audio alone (e.g., nodding, long pauses, and shaking heads). The observational notes of the researchers (CC, VS) supplemented the video to ensure accuracy of the transcript. After each interview, the PIs (CC, VS) would debrief and discuss salient points of the focus group. The recordings were then transcribed verbatim and reviewed by all the authors to check for accuracy. The data collection continued until saturation defined as a lack of new concepts or information collected from the participants was reached (Glaser & Strauss, 1967). An honorarium was provided to the participants in the form of an electronic gift card as a token of appreciation for their time.

### Data Analysis

**Figure 1.**
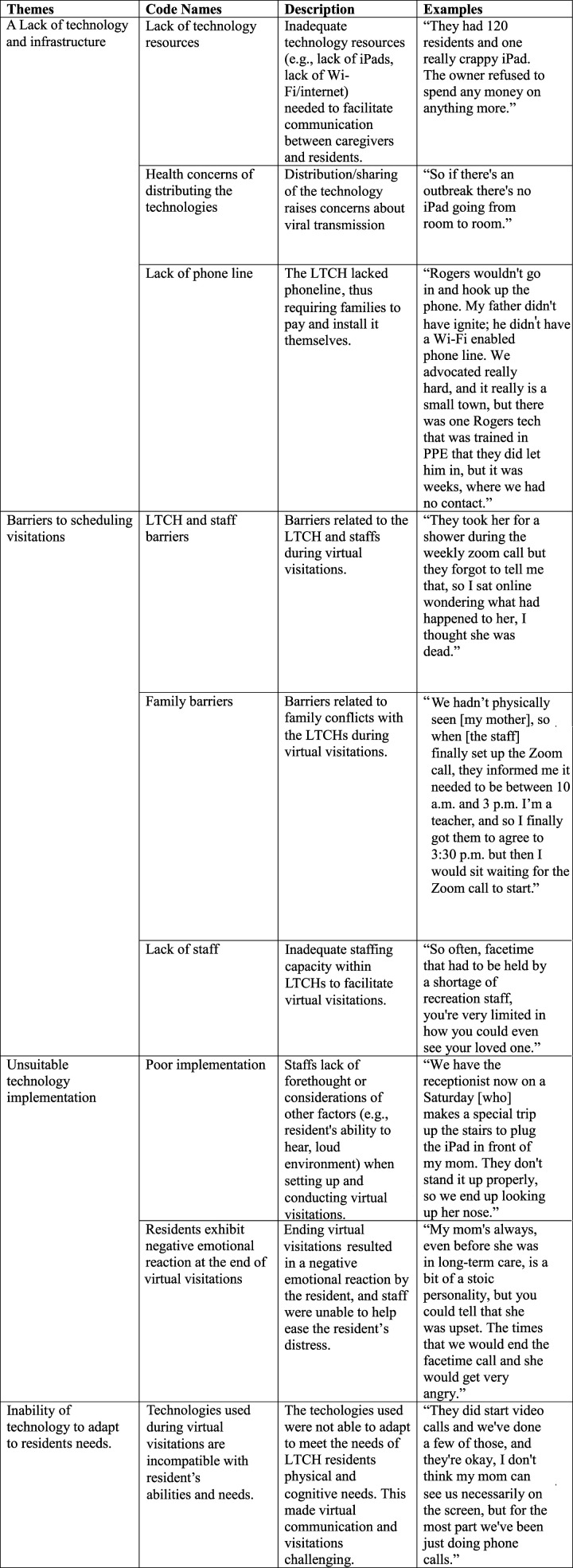
Coding Tree.

Braun & Clarke’s (Braun & Clarke, 2006) six-step process of thematic analysis was used to analyze the focus group interviews through NVivo 12 software. The six steps were as followes: 1) data familiarization; 2) developing the codes; 3) identifying the themes; 4) revising the themes; 5) describing the themes; and 6) writing the manuscript (Braun & Clarke, 2006). To get familiar with the data, one researcher (AY) listened and transcribed all audio recordings of the interviews. The PIs (CC, VS) wrote observational notes during the focus groups, which helped provide baseline codes during the line-by-line inductive thematic analysis. Later, the codes were grouped and labeled into relevant recurring themes and sub-themes which were discussed between the three researchers and refined. A coding dictionary was distributed across researchers to further refine the themes and their descriptors. The coding dictionary was checked by the PIs to iteratively provide feedback during regular biweekly meetings. Finally, a coding tree that outlines the thematic analysis was developed (see Figure 1).

## Results

Seven focus groups were conducted with 30 caregivers from Ontario and British Columbia, Canada. No interviews were repeated, and no participants dropped out.

### Participant demographics and characteristics

Table 1 provides a descriptive overview of the participants’ characteristics. All but one EFC was female (96%, *n* = 29). Caregivers were predominantly (76%, *n* = 23) the daughters of the LTCH residents. Most of the caregivers were between the ages of 55–64 (50%, *n* = 15) and employed (63%, *n* = 19). LTCH residents’ duration of stay in homes was commonly one to two years (46%, *n*=14) or three to five years (36%, *n* = 11). Most caregivers were caring for residents in “publicly owned (municipal)” (50%, *n* = 15) compared to “private, not for profit” (10%, *n* = 3) and “private, for profit” (26.7%, *n* = 8) LTCHs, and the majority of residents resided in private rooms (80%, *n* = 24).

**Table 1.** Descriptive characteristics of study participants (*n* = 30).

Characteristic	N (%)
Gender	
Female	29 (96.7)
Male	1 (3.3)
Age	
35–44	3 (10.0)
45–54	8 (26.7)
55–64	15 (50.0)
65+	4 (13.3)
Employed	
Yes	19 (63.3)
No	11 (36.7)
Relationship to LTCH resident	
Daughter	23 (76.7)
Son	1 (3.3)
Spouse	5 (16.7)
Grandchild	1 (3.3)
Resident’s length of stay	
<1 year	2 (6.7)
1–2 years	14 (46.7)
3–5 years	11 (36.7)
5+ years	3 (10.0)
Profit status of LTCH	
Private, not-for-profit	3 (10.0)
Private, for-profit	8 (26.7)
Publicly owned (municipal)	15 (50.0)
Unsure	4 (13.3)
Room type	
Private room	24 (80)
Semi-private room	6 (20)

Table 2 summarizes the responses of the EFCs to pre- and post-pandemic care and visitation questions or statements. Prior to COVID-19, most EFCs would visit their loved ones zero to three times a week (43%, *n* = 13), with visits lasting an average of 135 minutes. In response to the statement, “I was happy with the care my loved one was provided *before* the pandemic” most EFCs expressed that they either “agree” with the statement (30%, *n = 9*) or “neither agree nor disagree” (30%, *n = 9*). During COVID-19, most visits lasted an average of 81 minutes. Finally, over 60% of EFCs were dissatisfied with the care their loved ones received in LTCHs during COVID-19, with 33% (*n* = 10) “disagreeing” and 30% (*n* = 9 ) “strongly disagreeing” with the following statement, “I was happy with the care my loved one was provided during the pandemic”.

**Table 2.** Pre and post pandemic LTCH care and visitation (*N* = 30).

Question/Statement	N (%)
On average, how many times a week did you visit your loved one in LTCH before COVID-19?	
0–3 times per week	13 (43.3)
4–7 times per week	10 (33.3)
7+ per week	7 (23.3)
What was the average duration of your visits to the LTCH (in minutes) before COVID-19?	
Mean	135
Median/Mode	120
St. deviation	72.4
I was happy with the care my loved one was provided *before* the pandemic	
Agree	9 (30.0)
Strongly agree	4 (13.3)
Neither agree nor disagree	9 (30.0)
Strongly disagree	4 (13.3)
Disagree	4 (13.3)
What was the average duration of your visits to the LTCH (in minutes) during COVID-19?	
Mean	81
Median/mode	60
St. Deviation	75.6
I was happy with the care my loved one was provided during the pandemic	
Agree	5 (16.7)
Strongly agree	1 (3.3)
Neither agree nor disagree	5 (16.7)
Strongly disagree	9 (30.0)
Disagree	10 (33.3)

### Themes

The experiences of technology-based visitations among EFCs were organized into macro, meso, and micro-level factors: 1) a lack of technology and infrastructure; 2) barriers to scheduling visitations; 3) unsuitable technology implementation; and 4) inability of technology to adapt to residents' needs. We describe each theme below along with quotes with EFC psyudonyms or a randomly assigned number and which FG (e.g., FG1, FG2, FG3) they were in. 

### A Lack of Technology and Infrastructure

The lack of technological infrastructure and insufficient access to appropriate technology was a concern of EFCs who explained that their homes significantly lacked technological devices (e.g., iPads) such that there was not enough to share amongst all the residents:

“They had 120 residents and one really crappy iPad. The owner refused to spend any money on anything more” (EFC “Flow,” FG1).

It appeared that LTCH owners and administrators were reluctant to finance those resources (i.e., purchasing technology). As well, LTCHs often relied on donation drives which were unable to occur with COVID-19; consequently, any financial gaps were filled in by EFCs who often supplied their loved ones with the necessary technologies and/or wireless internet (Wi-Fi) networks:

“I even bought a tablet and I contributed money because they asked for people to donate so they could buy iPads for the facility.” (EFC “3,” FG6)

Beyond the already limited technology resources, some caregivers noted that virtual visitations were not conducted in the first wave of the pandemic due to the home’s hesitancy of COVID-19 transmission when sharing the devices across many residents—forcing some families to go months without seeing or hearing from their loved one in LTCHs:

“The [LTCH’s] recreation department said they didn’t have enough iPads, and then they’re concerned that people were gonna be touching the iPad. People not knowing [how COVID-19 was spreading] […] so they were afraid, but why [were other LTCHs] doing it? There are ways around that.” (EFC “1,” FG3)

The adoption of newer technology platforms that could allow EFCs to ascertain resident’s well-being and the conditions of care inside the home was met with resistance by LTCHs due to concerns over the potential lack of privacy and recording capabilities of the technology. In the unprecedented time of COVID-19, EFCs reported that the homes were more concerned about staff privacy than whether EFCs and residents could communicate :

“I installed something called an Alexa, a video phone in my husband’s room […] you don’t need to hit accept like you do on Skype or Facetime […] I could just [virtually] drop in […] [but] the [LTCH] was concerned that it might record the staff in the room. It doesn’t record.” (EFC “4,” FG6)

Some EFCs described how they were unable to have virtual visitation as the LTCH did not have the physical infrastructure (e.g., internet access) to support online visits: “this LTCH is older, they do not have Wi-Fi throughout the building. If you’re going to have Wi-Fi you need pay for it and it's only in your room.” (EFC “Queen,” FG1). In other instances, LTCH's attempts to cohort and isolate residents with COVID-19 meant that residents lost phone services when re-located to a new room. This demonstrated to residents and EFCs that the homes were not prioritizing quality of life or the facilitation of communication between residents and EFCs. One EFC described how her father was moved to a new COVID-19 unit but in doing so lost his his phone line and how difficult it was to get it reconnected to the new room due to infection control policies in place:

“[The LTCH] did offer facetime, but [my father] never held an iPad in his life, but it was good because my family members could see him at the same time. But then they moved him to a different floor in a different room saying that they needed to make a COVID-19 wing. So, when they moved him, and that was in the Spring 2020, he lost his phone. Rogers [the telephone company] wouldn’t go in and hook up the phone because of COVID-19. My father didn’t have [a phone line] – and he didn’t have Wi-Fi access. We advocated really hard…[eventually] one Rogers [telephone technician] that was trained in PPE that the home [let in], but it was weeks where we had no contact.” (EFC “1,” FG5)

### Barriers to Scheduling Visitations

EFCs reported a lack of coordination on the part of the LTCHs in facilitating the virtual visitations which was a significant barrier. A common occurrence was that LTCH staff would only schedule call-in video appointments during working hours (e.g., between 9 a.m. and 5 p.m.), leaving caregivers with limited options as most of them also worked full-time. The homes included in this study offered no accommodations for caregivers to video conference outside those hours; instead, caregivers recounted times where they needed to work around the LTCH’s hours of operation. Further, the process of scheduling and calling in was tedious due to the lack of staffing capacity to coordinate the video calls, leaving EFCs feeling frustrated and helpless when scheduled virtual visits did not occur as planned:

“We hadn’t physically seen [my mother], so when [the staff] finally set up the Zoom call, they informed me it needed to be between 10 a.m. and 3 p.m. I’m a teacher, and so I finally got them to agree to 3:30 p.m. but then I would sit waiting for the Zoom call to start. I’m still technically at work, probably 'till 4:30 to 5 o’clock, so I would sit there waiting and waiting and waiting and not doing my job, while I waited for this phone call that would never come.” (EFC “2,” FG4)

This was a common experience. EFCs would wait for days (up to a week) for their scheduled call to occur and often no one from the LTCH would pick up. A likely primary contributing factor was inadequate staffing levels in LTCHs during COVID-19. Specifically, ECFs frequently spoke about the heavy reliance on LTCH staff to set up virtual visits and with less staff there was a limited length and number of virtual visits offered to families:

“Facetime had to be held by a shortage of recreation staff, you’re very limited in how [long] you could even see your loved one.” (EFC “4,” FG6)

“I appreciate the restrictions for COVID-19, but the thing is my mom has seven children […] with the video calls, it’s like one call per resident per week and there’s seven of us [so most of us were unable to see her].” (EFC “4,” FG2)

### Unsuitable Technology Implementation

EFCs recalled feeling that the value of video conferencing was greatly diminished because staff inappropriately set up the technology—for example, the tablet was not properly positioned and/or the distance of the device from the resident’s face was too far. In the absence of dedicated staff to provide technical support; untrained frontline care staff had to manage the technical aspects of virtual visits alongside their care duties. Caregivers also experienced staff not preparing their loved ones for visitations, such as residents missing their hearing aids or holding the visitations in high traffic areas of the homes (e.g., noisy hallways), which limited the quality of the communication. Several EFCs described their experiences:

“[My mom] lost use of her hands entirely sometime over the summer, so she was now rendered quadriplegic. Then came the dependency on calling nurses at set times of day and being just dependent on their kindness, their time [and] their willingness to be an intermediary […] we did Facetime, but that was tough […] staff just not thinking, just holding it too far - she couldn’t see. She couldn’t hear, [it was the] wrong side of her face.” (EFC “Morgan,” FG1)

“She had a hearing aid, only one. They put it through the washing machine, the day before Christmas... [so she was not able to hear me on our call]” (EFC “Morgan,” FG1)

These experiences illuminate a lack of consideration regarding the environmental factors and physical/sensory impairments of residents preventing the proper use of these devices in the LTCH context. There seemed to be a lack of understanding by staff about how to use the technology, including the best conditions to video call, and its capabilities and limitations. EFCs expressed that virtual visits did not help facilitate connection with their loved ones and oftentimes led to further distress. EFCs witnessed how staff were unable to support residents through their feelings of frustration and agitation, which often followed the end of a video call:

“When the staff came to end the video calls, my mom became very aggressive with the staff. Not wanting to give back the iPad–not wanting to end the call […]. It was pretty brutal, actually, you can just see the emotion in terms of her loss of connection.” (EFC “3,” FG2)

“So, the facetime visits didn't really work very well. [My husband] got so upset and [he would] try and kiss the screen of the laptop, [making] it all slobbery […]. [He] did get a little bit agitated because he couldn’t touch me or see me or anything.” (EFC “4,” FG7)

Witnessing high levels of agitation from their loved ones due to loss of connection was an unpleasant and traumatic experience for both residents and EFCs to witness. Some EFCs made the difficult decision to discontinue virtual calls altogether out of guilt and the emotional toll of the video calls.

Furthermore, technology implementation was impeded by front line staff at the homes lacking adequate training to facilitate visitations. EFCs also reported that the lack of care staff on the unit necessitated other staff within the LTCHs (e.g., receptionist) to set up the technology. This problem was exacerbated during weekends and night shifts when these facilities are known to have even greater staffing shortages:

“We have the receptionist now on a Saturday [making] a special trip up the stairs to plug the iPad in front of my mom. They don't stand it up properly, so we end up looking up her nose but that’s okay. I’ll take that as opposed to nothing.” (EFC “2,” FG4)

Although video calls were not a substitute for in-person visitations and were poorly implemented, they were often the only option for EFCs to connect with their loved ones during the first year of the pandemic. The seperation was incredibly difficult for EFCs who were used to visiting their loved ones regularly, often multiple times a week. Despite the constraints, a few EFCs were grateful for simply a glimpse of their loved one: “I always looked forward to seeing [my husband] on Facetime even if just for 5 minutes. He sees me with no mask on and I guess […] he can still remember me.” (EFC “2,” FG2)

### Inability of Technology to Adapt to Residents’ Needs

EFCs know the challenges of communicating with their loved ones especially when there are cognitive and/or physical impairments. EFCs depend on LCTH staff to consider these impairments prior to using various devices for virtual visits but realized the ways that technology was not appropriate or suitable to meet residents’ needs (i.e., physical and/or cognitive impairments). In one instance, a family member used the video calls as an opportunity to visually assess the resident:

“[My mother] has macular degeneration, so I agree with [the other family members that] the Facetime video calling was useless because she can’t see. She can only hear us, and it was so noisy with [the] staff there.” (EFC “4,” FG5)

“For a 91-year-old with advanced dementia, [Skype is] just not an option; it wasn't an option for [my father], it was more for us so that I could see him and assess him. The first time we ever Skyped...I said [to the staff] “show me his back, show me his feet”, show me all the things that I was doing for him before we weren't allowed in.” (EFC “1,” FG3)

In sum, the LTCHs infrastructure with respect to devices, internet access, protocols, and staffing were not in place to support the demands for technology visits. Moreover the LTCHs overestimated the capability and suitability of technology to provide a meaningful connection between EFCs and the residents which consequently caused trauma to some EFCs and residents.

## Discussion

To our knowledge, this is the first descriptive qualitative study to examine the COVID-19 experiences of technology-based visitations among EFCs in LTCHs in Canada. Technology was often the only means of communication for caregivers to “visit” their loved ones during the lockdown policies, certainly in the first year of the pandemic when policies were most strict. While some caregivers appreciated the opportunity to connect virtually, there were barriers to technology implementation. Our findings demonstrate the important role of technology in connecting EFCs to their loved ones amidst visitor restrictions to LTCHs but conclude that the use of these technologies was challenged by the systemic and structural factors within LTCHs and the broader LTC system.

Importantly, our findings elucidate the complexity and potential *harms* of poorly implemented virtual visits. EFCs were faced with a myriad of concerns that included technical complications (e.g., “can my loved one log on to the internet?”), socio-emotional effects of the visit (e.g., “is my call going to upset my loved one?”) and physical aspects of care that may not be met (e.g., “I wonder if my loved one will have their hearing aid in for the visit”). These concerns contended with EFCs’ other feelings of frustration towards staff while appreciating the overburdened and underresourced LTCH context. Furthermore, when some families chose to stop virtual visits altogether, participants described the negative emotional toll on EFCs and residents. This was an incredibly painful and damaging decision for EFCs to make that was rife with shame and guilt. There appears to be a gap in understanding by decision-makers about how poor technology implementation contributes to potential harm. This work underscores the moral imperative to recognize the relational consequences of proposing solutions at a system level that cannot be properly implemented at a home level.

Furthermore, EFCs' dependancy on technology was reinforced by LTCHs that promoted the use of virtual visits but lacked the necessary resources, staff, and infrastructure. Our identified barriers were consistent with previous research including a lack of devices, lack of wireless internet access due to structural deficiencies (Eghtesadi, 2020), and lack of trained staff to support the use of technology—that is not designed with older adult use in mind (Barnard et al., 2013; Chu, Nyrup et al., 2021; Damodaran et al., 2014). The structural barriers within LTCHs are long-standing issues that were exacerbated during COVID-19 (Marrocco et al., 2021). Ageism and social exclusion of older people are likely contributing factors to these issues (Chu, Nyrup et al., 2021; World Health Organization, 2021). The absence of internet access for older adults residing in LTCHs is a failure of governments to ensure residents are connected to the world. This lack of internet access is contrary to the notion of access to the internet as a human right that is related to the right to freedom of expression, development, and assembly (La Rue, 2011). Provincial-level advocacy efforts from organizations, such as the Ontario Association of Residents’ Councils, have acknowledged that technology is vital for social connections among LTCH residents (Ontario Association of Residents’ Councils, 2020). While the Toronto government has provided only city-run LTCHs access to Wi-Fi (City of Toronto, 2020), these efforts remain temporary solutions. There is no federally mandated LTCH standard related to the accessibility of technology resources, internet, and Wi-Fi connectivity.

Technologies can enhance and facilitate relationships between EFCs, residents, and staff if there is adequate coordination and support. Unfortunately, our participants shared multiple accounts of scheduling issues, poor implementation, and lack of technology devices, which left them feeling helpless and excluded from care-related decision-making for a prolonged period of time. Consistent with our findings, a 2020 Patient Ombudsman report identified that the pandemic exacerbated poor staffing levels in LTCHs, which resulted in poor communication with families (Fooks, 2020). The understaffing in LTCHs led to instances of inadequate resident care; therefore, the additional demands for staff to facilitate virtual visitations were not possible in many cases (Ontario Health Coalition, 2020). Familiarity with the residents’ needs may be best approached through the consistent assignment of staff to facilitate stronger relationships with the residents and their families to engage in shared decision-making (Caspar et al., 2021; Chu, Quan, Gandhi, and McGilton, 2021), which was not possible during staffing crises. Resources and support are needed to shore up the structures of LTC to enable staff to collaborate with families. Literature indicated that collaboration can enhance decision-making processes, for example, engaging families in the development of visitations guidelines to use in the future (Canadian Foundation for Healthcare Improvement, 2020).

The rhetoric around various types of technologies as “care solutions” for the ageing population needs to be critically approached (Fischer et al., 2020; Lehoux & Grimard, 2018; Peek et al., 2014; Sixsmith & Gutman, 2013), for the fact that technologies as health interventions have the potential to create and exacerbate inequities of care through uneven access to services (Veinot et al., 2018). Technologically enabled visitations helped connect many families to their loved ones; however, access to devices or digital infrastructure was disproportionately distributed at a macro-level (i.e., within the LTC sector and healthcare system). The inequities can be addressed, for example, through policies to ensure that technological infrastructure is provided to all Canadian LTCHs and that technical training be provided to staff (e.g., personal support workers, nurses) regarding its proper implementation. Future research directions include exploring the exclusion of residents and families in technology design and implementation in LTCHs (Chu, Nyrup et al., 2021), as well as investigating the equity to access and distribution of technology and/or innovations in LTCH to inform a more inclusive future that involves the residents and families. Lastly, determining context and user-appropriate technology interventions, including features and design, that can support residents’ independent use of technology and enhance their ability to connect with families is warrented.

### Strength and Limitations

This study examined the experiences of caregivers from Ontario and British Columbia, Canada to provide a more comprehensive understanding of virtual visits. There are differences in the pandemic response between the provinces; however, the experiences of EFCs using technology to communicate with loved ones in LTCHs are likely to be similar and consistent. Therefore, our findings may be transferrable to other caregivers using technology in similar situations in different jurisdictions. The authors followed the Consolidated Criteria for Reporting Qualitative Studies (Tong et al., 2007) (Table S1) and generated a well-documented audit trail to ensure rigor, credibility, and transparency in this study. The PIs are aware that the positionality and subjectivity of the researcher are central to qualitative work. Both PIs were reflexive in how their different training (CC is a healthcare professional and holds a doctorate in Nursing, and VS has a doctorate in Sociology), occupations as professors in Ontario universities and personal experiences as female caregivers with family in LTCHs could have shaped the findings. The multidisciplinary aspect of this study can be considered a strength since different backgrounds can strengthen the design of a study and offer varying perspectives (Gale et al., 2013), and both PIs are researchers with in-depth knowledge of the LTCH sector. A potential limitation is that recruitment may have only included individuals who were interested in LTCHs. There is a possibility that dissatisfied caregivers were more motivated to participate in this research; however, the sample was from two provinces in Canada, included a wide age range of caregivers (29–70 years old) with loved ones in public, private, for-profit and not-for-profit LTCHs. Given the diversity in the sample, our findings with poor virtual visitations during COVID-19 may be more commonplace rather than an overrepresentation of dissatisfied EFCs as other Canadian studies about family caregivers also report negative experiences (Badone, 2021; Dupuis-Blanchard et al., 2021; Hindmarch et al., 2021). Finally, the study’s participants were mostly female and Caucasian/European. Future research should consider examining EFCs experiences within other provinces and include other ethnic groups to capture their experiences.

## Conclusion

The infection control public health policies that restricted all visitors from LTCHs in an attempt to protect residents from COVID-19 created a dependency on technologies for virtual visitations between EFCs and their loved ones. This qualitative study provided insight into the experiences and challenges of those visitations at multiple levels (micro, meso, and macro). Our study highlighted that more thoughtful approaches are needed to enable EFCs and residents to maintain their relationships during periods of restricted visitations and to mitigate any long-term trauma and harm. There are multiple issues that need to be addressed at proximal and distal levels that prevented LTCHs from utilizing technology to its fullest potential to promote person-centered care post-pandemic.

## Supplemental Material

sj-pdf-1-jag-10.1177_07334648221081850 – Supplemental Material for Poor and Lost Connections: Essential Family Caregivers’ Experiences Using Technology with Family Living in Long-Term Care Homes during COVID-19 Click here for additional data file. Supplemental Material, sj-pdf-1-jag-10.1177_07334648221081850 for Poor and Lost Connections: Essential Family Caregivers’ Experiences Using Technology with Family Living in Long-Term Care Homes during COVID-19 by Charlene H. Chu, Amanda V. Yee and Vivian Stamatopoulos in Journal of Applied Gerontology
