# Supplementary material for: Poor and Lost Connections: Essential Family Caregivers’ Experiences Using Technology with Family Living in Long-Term Care Homes during COVID-19
Source: J Appl Gerontol. 2022 Apr 13;41(6):1547–56. doi: 10.1177/07334648221081850 (PMC9014337; doi:10.1177/07334648221081850)
Supplement: sj-pdf-1-jag-10.1177_07334648221081850 – Supplemental Material for Poor and Lost Connections: Essential Family Caregivers’ Experiences Using Technology with Family Living in Long-Term Care Homes during COVID-19 [file sj-pdf-1-jag-10.1177_07334648221081850.pdf]

Table S1: Consolidated criteria for Reporting Qualitative research (COREQ) Checklist

No. Item	Guide questions/description	Answers	page
**Domain 1: Research team and reflexivity**			
Personal Characteristics			
1. Interviewer/facilitator	Which author/s conducted the interview or focus group?	CC; VS	2
2. Credentials	What were the researcher's credentials? E.g. PhD, MD	CC is an RN, GNC(c), PhD; VS is a PhD	7
3. Occupation	What was their occupation at the time of the study?	Both PIs are professors at Ontario universities.	7
4. Gender	Was the researcher male or female?	Both PIs are female.	7
5. Experience and training	What experience or training did the researcher have?	Both PIs are well versed in qualitative research and have published papers using qualitative methods.	7
Relationship with participants			
6. Relationship established	Was a relationship established prior to study commencement?	No. Some participants may have been familiar with the PIs' work due to advocacy efforts. The PIs did not establish personal relationships with the participants.	2
7. Participant knowledge of the interviewer	What did the participants know about the researcher? e.g. personal goals, reasons for doing the research	CC is a researcher focused on improving the care of older adults in LTC and VS is a prominent advocate for and researcher of LTCH families. Both have in-depth knowledge about LTCH care (e.g. CC conducted her PhD in LTCHs).	7
8. Interviewer characteristics	What characteristics were reported about the interviewer/facilitator? e.g. Bias, assumptions, reasons and interests in the research topic	Both interviewers, CC and VS, have expertise in qualitative methods by background and training.	7
**Domain 2: study design**			
Theoretical framework			
9. Methodological	What methodological orientation was	Thematic analysis.	2-3

orientation and Theory	stated to underpin the study? e.g. grounded theory, discourse analysis, ethnography, phenomenology, content analysis		
Participant selection			
10. Sampling	How were participants selected? e.g. purposive, convenience, consecutive, snowball	Purposive sampling, with the intent to recruit more male caregivers to have equal numbers of males and females, but we were not able to.	2
11. Method of approach	How were participants approached? e.g. face-to-face, telephone, mail, email	Social media (i.e., Twitter) was used to recruit participants. Interested participants emailed the PIs and communication occurred via email.	2
12. Sample size	How many participants were in the study?	30	3
13. Non-participation	How many people refused to participate or dropped out? Reasons?	0	3
Setting			
14. Setting of data collection	Where was the data collected? e.g. home, clinic, workplace	Virtually over Zoom, a video conferencing platform.	2
15. Presence of non-participants	Was anyone else present besides the participants and researchers?	No.	2
16. Description of sample	What are the important characteristics of the sample? e.g. demographic data, date	All were family caregivers of a loved one living in LTCH and were unable to visit for extended periods of time during the COVID-19 pandemic. These family members considered themselves “essential caregivers” and provided emotional support and companionship for their loved ones. Characteristics of the EFCs and their loved ones are provided in a table.	2 and table 1
Data collection			
17. Interview guide	Were questions, prompts, guides provided by the authors? Was it pilot tested?	Yes, a pilot-tested, semi-structured interview guide was used.	2
18. Repeat interviews	Were repeat interviews carried out? If yes, how many?	No.	3
19. Audio/visual recording	Did the researcher use audio or visual recording to collect the data?	Video recording.	2

20. Field notes	Were field notes made during and/or after the interview or focus group?	Yes, field notes were made. The PIs discussed them after each focus group. These also served to inform the initial generation of the codes.	2-3
21. Duration	What was the duration of the interviews or focus group?	90 minutes for each focus group.	2
22. Data saturation	Was data saturation discussed?	Yes.	2
23. Transcripts returned	Were transcripts returned to participants for comment and/or correction?	No.	N/A
**Domain 3: analysis and findings**			
Data analysis			
24. Number of data coders	How many data coders coded the data?	Three; AY coded all the transcripts and the coding dictionary was checked by the PIs (CC and VS), to refine the codes and the development of the themes.	3
25. Description of the coding tree	Did authors provide a description of the coding tree?	Yes.	Figure 1
26. Derivation of themes	Were themes identified in advance or derived from the data?	Inductive; themes were derived from the data.	2-3
27. Software	What software, if applicable, was used to manage the data?	NVivo 12 software.	3
28. Participant checking	Did participants provide feedback on the findings?	No.	N/A
Reporting			
29. Quotations presented	Were participant quotations presented to illustrate the themes/findings? Was each quotation identified? e.g. participant number	Yes, quotes were used. Alongside the quotes, are the anonymized name or number and the focus group number that the participant was in.	4-6
30. Data and findings consistent	Was there consistency between the data presented and the findings?	Yes.	4-6
31. Clarity of major themes	Were major themes clearly presented in the findings?	Yes.	4-6

32. Clarity of minor themes	Is there a description of diverse cases or discussion of minor themes?	Yes.	4-6

Developed from: Tong A, Sainsbury P, Craig J. Consolidated criteria for reporting qualitative research (COREQ): a 32-item checklist for interviews and focus groups. International Journal for Quality in Health Care. 2007; 19, 349 – 357.
